# Relationship Between Facial Motor Impairment, Swallowing Function and Oral Motor Dysfunction in Patients with Post-Stroke Central Facial Paralysis

**DOI:** 10.3390/diagnostics16121787

**Published:** 2026-06-10

**Authors:** Burak Manay, Ramazan Güven, Alperen Şentürk, Mustafa İbas

**Affiliations:** 1 Department of Speech and Language Therapy, Faculty of Health Sciences, Istanbul Atlas University, Istanbul 34403, Turkey; alperen.senturk@atlas.edu.tr; 2 Department of Emergency Medicine, Faculty of Medicine, Istanbul Atlas University, Istanbul 34403, Turkey; 3 Department of Otolaryngology—Head and Neck Surgery, Istanbul Atlas University, Istanbul 34403, Turkey

**Keywords:** stroke, central facial paralysis, dysphagia, oral motor function, swallowing

## Abstract

**Background and Objectives:** Central facial paralysis following stroke may affect oral motor control and swallowing function; however, its role in dysphagia-related impairments remains unclear. This study aimed to investigate the relationship between facial motor impairment, swallowing function, and oral motor dysfunction in patients with post-stroke central facial paralysis. **Methods:** This cross-sectional observational study included 80 patients with ischemic or hemorrhagic stroke accompanied by central facial paralysis. Facial motor function was evaluated using the Sunnybrook Facial Grading System. Swallowing function was assessed using the Eating Assessment Tool-10 (EAT-10), Repetitive Saliva Swallowing Test (RSST), and Functional Oral Intake Scale (FOIS). Oral motor dysfunction was evaluated using a clinician-based oral motor assessment form. Correlation and exploratory regression analyses were performed. **Results:** Significant associations were identified between facial motor function, swallowing measures, and oral motor parameters (all *p* < 0.001). Sunnybrook scores showed strong negative correlations with EAT-10 scores (r = −0.954) and positive correlations with RSST (r = 0.914) and FOIS scores (r = 0.915). In exploratory regression analyses, NIHSS emerged as an important contributor to swallowing-related outcomes. Labial and tongue functions were independently associated with RSST performance, whereas labial, velar, and buccal functions were independently associated with EAT-10 scores. Velar function was the only oral motor variable independently associated with FOIS levels. **Conclusions:** Facial motor function, oral motor dysfunction, and swallowing-related measures were significantly associated in individuals with post-stroke central facial paralysis. Overall, neurological severity also contributed substantially to swallowing-related outcomes. These findings may suggest a potential association between oral motor dysfunction and swallowing-related outcomes and may warrant consideration in comprehensive dysphagia evaluation. However, the observed relationships should be interpreted as exploratory and require confirmation in future studies using validated assessment tools and instrumental swallowing measures.

## 1. Introduction

Stroke is a heterogeneous neurological condition that may affect different regions of the central nervous system, and its clinical manifestations are not limited to extremity motor dysfunction. One of the commonly observed findings following stroke is central facial paralysis (CFP), which is characterized by impaired voluntary and involuntary motor control of the facial muscles. Although CFP is generally addressed in clinical practice in relation to speech and facial expression, its effects on oral motor functions involved in swallowing and feeding are frequently overlooked [1].

The facial nerve plays a critical role in lip closure, oral competence, and buccal stability, particularly through the orbicularis oris and buccinator muscles. Weakness or asymmetry in these muscle groups may lead to anterior oral leakage, buccal residue accumulation, impaired bolus control, and the development of compensatory strategies during mastication [1,2]. Therefore, facial paralysis should be considered not only an aesthetic problem but also a functional disorder directly affecting feeding behaviors.

The relationship between facial paralysis and swallowing or oral motor functions has been predominantly investigated in individuals with peripheral facial paralysis, Bell’s palsy, or postsurgical facial nerve injury. Previous studies have reported increased dysphagia frequency, impaired bolus control, and widespread oral residue formation in these populations [1,2,3]. However, the extent to which these findings can be generalized to stroke populations with CFP remains unclear.

Although CFP following a stroke may directly affect oral phase functions, this topic has been relatively underexplored in the literature. Weakness of the lip and cheek muscles may significantly impair mastication efficiency and bolus control, potentially increasing the risk of dysphagia [4]. Nevertheless, the extent to which post-stroke dysphagia is associated with facial paralysis-related oral motor components has not been comprehensively evaluated in most studies.

Recent evidence suggests that post-stroke dysphagia should be considered a multidimensional disorder involving not only pharyngeal swallowing safety but also oral motor performance, feeding efficiency, nutritional status, and functional oral intake. Furthermore, growing attention has been directed toward the contribution of orofacial dysfunction to swallowing-related impairments following stroke, highlighting the importance of comprehensive clinical assessments that include oral motor and facial motor functions in addition to traditional dysphagia measures [5,6].

In addition, most currently available assessment approaches focus primarily on the pharyngeal phase or involve instrumental and imaging-based methods. In contrast, studies investigating facial paralysis-specific oral motor dysfunction using clinical, bedside, and non-invasive approaches remain limited [7]. This limitation may contribute to the insufficient understanding of the specific contribution of CFP to swallowing dysfunction, particularly in stroke populations.

Recent reviews have also emphasized that speech, swallowing, and oral competence problems associated with facial paralysis may significantly affect functional outcomes and quality of life. However, studies specifically examining the relationship between central facial paralysis, oral motor dysfunction, and swallowing function in stroke populations remain scarce [5,8].

This study aimed to investigate the clinical profile of swallowing function and oral motor dysfunction in individuals with post-stroke CFP using non-invasive assessment methods. The primary objective was to examine the relationship between facial paralysis severity, dysphagia presence, swallowing function, and oral motor components.

## 2. Methods

### 2.1. Study Design and Setting

This study was conducted as a cross-sectional observational clinical study investigating the relationship between facial function severity, swallowing function, functional oral intake level, and oral motor functions in individuals with post-stroke facial paralysis. Ethical approval was obtained from the Non-Interventional Scientific Research Ethics Committee of Istanbul Atlas University (Decision No: 01/14, 26 January 2026).

The study was carried out at the Neurology and Physical Medicine and Rehabilitation clinics of Istanbul Atlas University Hospital. Adult individuals with clinically and/or radiologically confirmed ischemic or hemorrhagic stroke accompanied by post-stroke facial paralysis were included in the study. Participants were recruited using a convenience sampling method, and written informed consent was obtained from all participants prior to study participation.

Data were collected prospectively using non-invasive clinical and functional assessment methods. Facial motor function was evaluated using the Sunnybrook Facial Grading System. Swallowing assessments included the Eating Assessment Tool-10 (EAT-10), Repetitive Saliva Swallowing Test (RSST), and Functional Oral Intake Scale (FOIS). Oral motor functions were evaluated using a brief clinician-based oral motor assessment form. In addition, clinical variables including age, stroke severity, stroke side, and time since stroke were recorded and considered as potential confounding variables in the analyses.

### 2.2. Study Population

**Figure 1:**
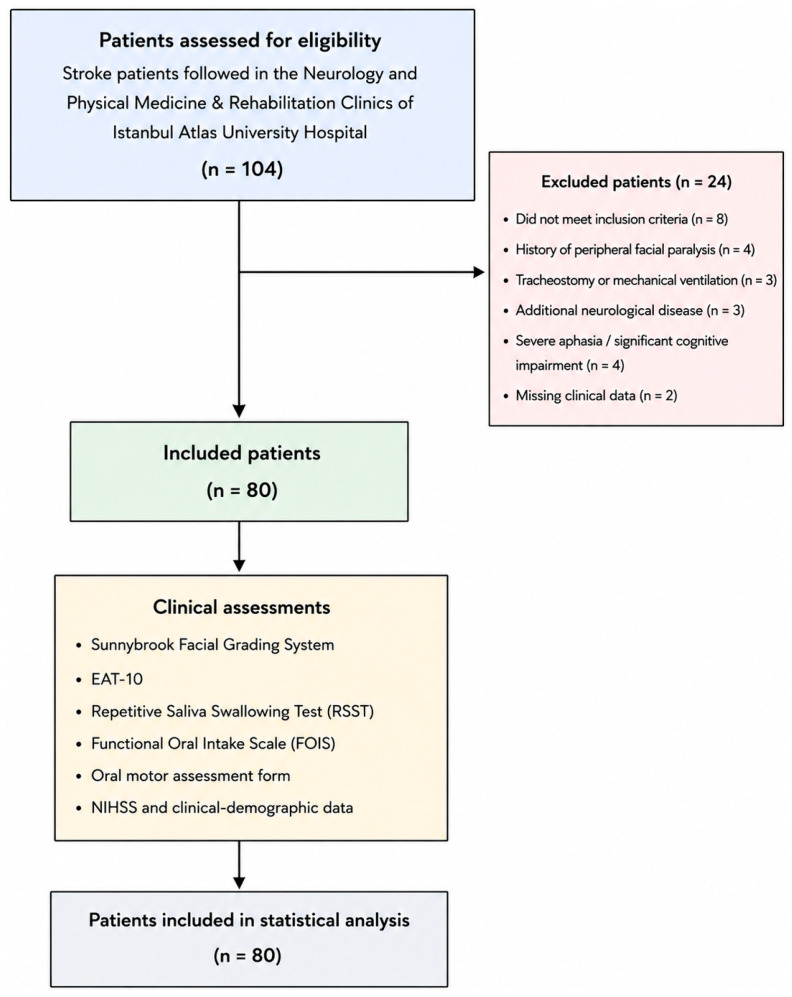
Flowchart of participant selection and study inclusion process.

The study population consisted of adult individuals followed at the Neurology and Physical Medicine and Rehabilitation clinics of Istanbul Atlas University Hospital with clinically and/or radiologically confirmed ischemic or hemorrhagic stroke diagnoses. A total of 80 patients with post-stroke CFP were included in the study. A flowchart illustrating the participant selection process is presented in Figure 1.

The presence of CFP was determined based on neurological examination findings and clinically/radiologically confirmed stroke diagnosis. CFP was defined as contralateral facial weakness predominantly affecting the lower facial muscles, with relative preservation of forehead muscle function, consistent with supranuclear facial involvement. Individuals presenting with findings suggestive of peripheral facial paralysis, including complete unilateral facial involvement, history of Bell’s palsy, facial nerve surgery, or peripheral facial nerve injury, were excluded from the study.

Participants were recruited using a convenience sampling method, and written informed consent was obtained from all participants prior to study inclusion. Only individuals aged 18 years or older, at least one month post-stroke onset, clinically stable, and with sufficient cognitive and communicative abilities to understand and respond to the assessment tools were included.

Individuals with a history of dysphagia prior to stroke, head and neck cancer, head and neck surgery, or additional neurological disorders that could affect swallowing and oral motor functions, such as Parkinson’s disease, Amyotrophic Lateral Sclerosis (ALS), or advanced dementia, were excluded. Patients with tracheostomy, requirement for mechanical ventilation support, severe aphasia, or severe cognitive impairment preventing compliance with the assessments were also excluded from the study.

### 2.3. Data Sources

Research data were collected through face-to-face clinical assessments, researcher observations, and clinical information obtained from the electronic medical record system. All assessments were performed using non-invasive methods.

Demographic and clinical characteristics of the participants, including age, sex, stroke type, stroke side, time since stroke, and stroke severity, were obtained using a data collection form prepared by the researchers. Stroke diagnoses were confirmed according to the International Classification of Diseases, 10th Revision (ICD-10) codes recorded in the electronic medical record system. Ischemic stroke diagnoses were primarily identified using I63 codes, whereas hemorrhagic stroke diagnoses were identified using I61 and I62 codes. Stroke severity was evaluated using the National Institutes of Health Stroke Scale (NIHSS). NIHSS scores were reassessed by the researcher on the evaluation day to reflect the participants’ current neurological impairment level.

Facial motor function was evaluated using the Sunnybrook Facial Grading System. This system is a clinical assessment tool that includes resting symmetry, symmetry of voluntary movements, and synkinesis components, with total scores ranging from 0 to 100. Higher scores indicated better facial motor function.

EAT-10 was used to subjectively evaluate dysphagia symptom severity. Functional oral intake level was assessed using FOIS, whereas RSST was applied as a clinical swallowing screening tool.

Oral motor functions were evaluated using a brief clinician-based oral motor assessment form developed by the researchers. This assessment included clinical parameters related to labial function, tongue function, mandibular control, velar function, buccal control, and oral motor behaviors. The internal consistency and inter-rater reliability of the oral motor assessment form were statistically analyzed.

### 2.4. Assessment Procedures

All clinical assessments were performed under standardized clinical conditions by the same researcher experienced in dysphagia and neurological rehabilitation. This approach was preferred to ensure assessment standardization across participants. Evaluations were conducted during a clinically stable period and within the same assessment session for each participant. First, demographic and clinical data were recorded, followed by facial motor function assessment using the Sunnybrook Facial Grading System, oral motor assessment, and swallowing assessments including EAT-10, RSST, and FOIS, which were administered in a standardized order.

During the assessment process, participants’ clinical stability was monitored, and short rest periods were provided when necessary to minimize fatigue or factors that could affect performance. To evaluate the inter-rater reliability of the oral motor assessment form, assessments were independently rescored by a second researcher, and the intraclass correlation coefficient (ICC) was calculated.

#### 2.4.1. Facial Function Assessment (Sunnybrook Facial Grading System)

Facial motor function was evaluated using the Sunnybrook Facial Grading System. The Sunnybrook system is a widely used clinical facial assessment tool that evaluates resting symmetry, symmetry of voluntary facial movements, and the presence of synkinesis. Total scores range from 0 to 100, with higher scores indicating better facial motor function. Assessments were performed during standard clinical examinations in all participants included in the study. Although the Sunnybrook Facial Grading System was originally developed and validated for peripheral facial palsy, recent evidence has demonstrated acceptable reliability in patients with stroke-related central facial palsy, supporting its potential applicability in this population [9]. Furthermore, the Turkish version of the scale has demonstrated satisfactory validity and reliability properties [10].

#### 2.4.2. Swallowing Assessment

Swallowing function was evaluated using EAT-10, RSST, and FOIS.

EAT-10 is a unidimensional self-report scale consisting of 10 items developed by Belafsky et al. (2008) [11]. EAT-10 was administered to evaluate the perceived severity of dysphagia symptoms. Each item is scored between 0 and 4, with higher total scores indicating greater dysphagia symptom severity. The Turkish validity and reliability study of the scale was conducted by Demir et al. (2016) [12], who reported a Cronbach’s alpha coefficient of 0.91. EAT-10 evaluates the perceived severity of swallowing-related symptoms in adults [12].

RSST was applied as a clinical swallowing screening method. Participants were instructed to swallow their saliva as many times as possible within 30 s, and the number of swallows during this period was recorded. Laryngeal movements were evaluated through clinical observation during the test. Fewer than three swallows within 30 s was considered indicative of swallowing impairment risk [13]. The number of swallows was recorded by the researcher through palpation of laryngeal elevation and clinical observation.

Functional oral intake level was evaluated using FOIS. FOIS is a widely used functional clinical scale designed to evaluate an individual’s current oral intake capacity and need for enteral feeding support. The scale classifies oral intake levels from 1 to 7, with lower scores indicating limited oral intake and higher scores indicating full oral intake [14].

EAT-10, RSST, and FOIS were selected because they provide complementary information regarding perceived dysphagia symptoms, bedside swallowing performance, and functional oral intake, respectively. Together, these tools allow a multidimensional clinical evaluation of swallowing function without requiring instrumental assessment.

#### 2.4.3. Oral Motor Function Assessment

Oral motor functions were evaluated using a brief clinician-based Oral Motor Dysfunction (OMD) assessment form developed by the researchers. The assessment included clinical parameters related to labial function, tongue function, mandibular control, velar function, buccal control, and oral motor behaviors. Assessment parameters were developed based on key structures involved in oral phase swallowing physiology and reported to be affected by facial paralysis. In particular, the effects of labial closure, buccal stability, lingual transport, and velopharyngeal control on bolus organization and oral transfer were considered.

Labial function assessment included resting tone, voluntary lip closure, lip movements, and anterior oral control behaviors. Tongue function assessment included protrusion, lateralization, and clinical tongue strength evaluation. Mandibular control assessment included opening-closing control, mandibular stability, and mastication pattern. In addition, velar elevation, signs of nasal leakage, and buccal residue behaviors were evaluated through clinical observation. Buccal control assessment specifically included food accumulation tendency in the oral vestibule, residue behavior within the buccal sulcus, and oral content control.

Each subcomponent of the oral motor assessment form was scored according to clinical performance level. Labial and tongue functions were scored between 0 and 8 points, mandibular control between 0 and 6 points, and velar and buccal functions between 0 and 4 points. Higher scores reflected poorer oral motor dysfunction. A total OMD score was calculated by summing the subcomponent scores, with higher scores reflecting greater oral motor dysfunction.

The scoring ranges were determined according to the number and clinical complexity of observable tasks included within each oral motor domain. Domains requiring evaluation of a greater number of functional components (e.g., labial and tongue functions) were assigned wider scoring ranges, whereas domains involving fewer clinically observable parameters (e.g., velar and buccal functions) were assigned narrower ranges. Because the OMD form was developed as an exploratory clinical assessment tool, these scoring weights were based on clinical judgment and functional relevance rather than on a formal psychometric weighting procedure.

All evaluations were performed during standard clinical examinations, and no invasive procedures, food administration, or experimental interventions were applied. The internal consistency and inter-rater reliability of the oral motor assessment form were statistically analyzed. This form was developed by the researchers to provide a brief clinical evaluation of oral motor impairment associated with CFP, based on key structures involved in oral phase swallowing physiology. Existing oral motor assessment approaches and clinical observation parameters were considered during the development of the form.

To the best of our knowledge, there is currently no widely accepted, validated clinical assessment tool specifically designed to evaluate oral motor impairments associated with CFP in adult stroke populations. Therefore, the present clinician-based oral motor assessment form was developed to evaluate oral preparatory and oral motor components potentially related to swallowing function in this population.

### 2.5. Statistical Analysis

Statistical analyses were performed using IBM SPSS Statistics software (Version 30.0; IBM Corp., Armonk, NY, USA). Descriptive statistics were calculated for all variables. Continuous variables were expressed as mean ± standard deviation or median (minimum–maximum) according to data distribution, while categorical variables were presented as frequency and percentage (%).

The normality of continuous variables was assessed using the Shapiro–Wilk test. Parametric analyses were applied for normally distributed variables, whereas non-parametric analyses were used for variables that did not meet normality assumptions.

Associations between facial motor function severity (Sunnybrook score), swallowing function, RSST, FOIS, EAT-10, and OMD parameters were evaluated using Pearson or Spearman correlation analyses according to variable distribution characteristics.

The internal consistency of the OMD assessment form was evaluated using Cronbach’s alpha coefficient. Inter-rater reliability was assessed using the intraclass correlation coefficient (ICC) based on a two-way random-effects model with absolute agreement.

Hierarchical regression analyses were performed to explore the associations between oral motor dysfunction parameters and swallowing-related outcomes. For RSST and EAT-10 outcomes, two-step hierarchical multiple linear regression models were constructed. In Model 1, demographic and clinical variables (age, sex, stroke type, stroke side, time since stroke, and NIHSS score) were entered as control variables. In Model 2, oral motor function variables (labial function, tongue function, mandibular function, velar function, and buccal control) were additionally entered to examine their contribution beyond general clinical characteristics.

Because FOIS is an ordinal clinical scale, ordinal logistic regression analysis was performed instead of linear regression. Similar to the hierarchical regression approach, demographic and clinical variables were entered in the first model, followed by oral motor function variables in the second model.

Sunnybrook Facial Grading System scores were evaluated separately in correlation analyses and descriptive assessments. To avoid conceptual overlap between global facial motor function and oral motor subdomain measures, Sunnybrook scores were not entered simultaneously with oral motor dysfunction subdomain variables in the primary regression models.

Before regression analyses, multicollinearity among predictors was assessed using the Variance Inflation Factor (VIF) values. Because some predictors reflected related aspects of post-stroke motor impairment, VIF values were carefully examined, and findings were interpreted cautiously where evidence of multicollinearity was present. Model assumptions, including normality and homoscedasticity of residuals, were evaluated before analysis.

Given the cross-sectional and exploratory nature of the study, no formal a priori sample size calculation was performed. Furthermore, because several outcome measures represented ordinal or semi-quantitative clinical scales, regression analyses were intended to explore potential associations rather than establish predictive models or causal relationships. Therefore, all regression findings should be interpreted as exploratory. Statistical significance was set at *p* < 0.05 for all analyses.

## 3. Results

### 3.1. Participant Characteristics

A total of 80 patients with post-stroke central facial paralysis were included in the study. The sample consisted of 41 females (51.2%) and 39 males (48.8%). Ischemic stroke was slightly more common than hemorrhagic stroke, and most participants had unilateral stroke involvement.

The mean age was 53.73 ± 15.73 years, and the mean time since stroke was 7.53 ± 6.61 months. The mean NIHSS score was 14.61 ± 13.68, while the mean Sunnybrook Facial Grading System score was 59.79 ± 31.79.

Swallowing and oral motor assessments demonstrated varying degrees of impairment across participants. The mean EAT-10, RSST, and FOIS scores were 16.76 ± 11.37, 5.83 ± 3.02, and 4.21 ± 1.95, respectively. Oral motor assessment findings indicated impairment in labial, lingual, mandibular, velar, and buccal functions. Detailed participant characteristics are presented in Table 1.

#### Oral Motor Assessment Reliability

Since the oral motor assessment form used in this study was developed by the researchers for clinical observational purposes, internal consistency and inter-rater reliability analyses were performed to evaluate the measurement reliability of the tool.

The overall internal consistency of the form was good, with a Cronbach’s alpha coefficient of 0.859. Inter-rater reliability analysis demonstrated moderate agreement for single measurements (ICC = 0.549, 95% CI: 0.449–0.649) and high agreement for average measurements (ICC = 0.859, 95% CI: 0.803–0.902) (*p* < 0.001).

Detailed correlation analysis results are presented in Table 2. Spearman correlation analysis demonstrated significant associations between facial motor function, swallowing measures, and oral motor parameters (all *p* < 0.001). Sunnybrook Facial Grading System scores showed a strong negative correlation with EAT-10 scores (r = −0.954) and strong positive correlations with RSST (r = 0.914) and FOIS scores (r = 0.915).

Similarly, total oral motor dysfunction (OMD) scores demonstrated strong correlations with EAT-10 (r = 0.936), RSST (r = −0.871), and FOIS (r = −0.915). Among oral motor domains, labial and tongue functions showed the strongest associations with swallowing-related measures. Overall, higher Sunnybrook scores and lower OMD scores were associated with better RSST and FOIS scores and lower EAT-10 scores.

To further evaluate factors associated with swallowing-related outcomes beyond baseline clinical characteristics, hierarchical regression analyses were performed for RSST and EAT-10, whereas hierarchical ordinal logistic regression analysis was conducted for FOIS. For the RSST outcome, NIHSS score was the only significant predictor in Model 1 (β = −0.691, *p* < 0.001). After oral motor variables were added in Model 2, NIHSS remained significant (β = −0.381, *p* = 0.031). Labial function (β = −0.343, *p* = 0.016) and tongue function (β = 0.246, *p* = 0.038) emerged as independent factors associated with RSST performance, whereas mandibular function, velar function, and buccal control were not significantly associated with the outcome (all *p* > 0.05).

For EAT-10 scores, NIHSS was the only significant clinical predictor in Model 1 (β = 0.974, *p* < 0.001). In Model 2, NIHSS remained significantly associated with EAT-10 scores (β = 0.551, *p* < 0.001). In addition, labial dysfunction (β = 0.266, *p* = 0.003), velar dysfunction (β = 0.110, *p* = 0.007), and impaired buccal control (β = 0.123, *p* = 0.047) were independently associated with higher EAT-10 scores. A summary of the significant predictors identified in the hierarchical regression analyses for RSST and EAT-10 outcomes is provided in Table 3.

For FOIS, hierarchical ordinal logistic regression analysis demonstrated that longer time since stroke (OR = 0.744, *p* = 0.013) and higher NIHSS scores (OR = 0.799, *p* < 0.001) were associated with lower functional oral intake levels in Model 1. After oral motor variables were entered into Model 2, velar dysfunction emerged as the only independent factor associated with FOIS levels (OR = 0.357, *p* < 0.001), whereas the remaining oral motor variables were not significantly associated with functional oral intake after adjustment. A summary of the significant predictors identified in the hierarchical ordinal logistic regression analysis for FOIS is provided in Table 4.

Multicollinearity diagnostics demonstrated elevated VIF values for several predictors, particularly variables reflecting overall stroke severity and related functional impairments.

## 4. Discussion

In this study, we explored the relationships among facial motor function, oral motor dysfunction, and swallowing-related measures in individuals with post-stroke CFP. Significant associations were observed between facial motor function, oral motor parameters, and both subjective and functional swallowing outcomes. However, the regression analyses showed that overall neurological severity, as reflected by NIHSS scores, remained an important contributor to swallowing-related outcomes. Therefore, the observed relationships should be interpreted cautiously within the broader context of post-stroke neurological impairment and should not be considered evidence of isolated effects of facial motor dysfunction.

This finding is consistent with previous studies reporting that greater neurological impairment is associated with increased dysphagia risk and poorer oropharyngeal swallowing function after stroke [15]. In addition, the strong correlations observed between facial motor, oral motor, and swallowing-related variables may reflect, at least in part, overlapping clinical constructs and shared functional mechanisms involved in oral phase swallowing control.

In our study, the strong associations between buccal control, labial function, and swallowing may suggest that oral phase organization may be associated with CFP-related oral motor impairments. In particular, impaired lip closure and reduced buccal stability may negatively affect oral bolus control and contribute to dysphagia symptoms. The facial nerve plays an important role in oral competence, lip closure, and buccal stability through the orbicularis oris and buccinator muscles. Previous studies have reported that weakness in these muscle groups may lead to anterior oral leakage, oral vestibular residue, reduced oral clearance, and impaired bolus control within the oral cavity [8]. Recent evidence has further highlighted that facial paralysis may adversely affect oral competence, oral residue management, and bolus control, contributing to swallowing-related difficulties [8]. Orofacial dysfunction following stroke has also been reported to affect not only speech and facial expressions but also oral control, bolus organization, and swallowing function [5]. Our findings are consistent with these studies. Recent evidence has demonstrated that facial palsy, difficulty blowing the cheeks, tongue movement disorders, and dysarthria are associated with dysphagia severity in acute ischemic stroke. These findings suggest that impairments involving facial, lingual, and oral motor functions may contribute to oral phase swallowing dysfunction. The results of the present study further support the importance of oral motor impairments in swallowing-related difficulties among individuals with post-stroke central facial paralysis [16]. These findings are consistent with emerging evidence indicating that facial paralysis may contribute to oral incompetence, oral residue, impaired bolus control, dysarthria, and dysphagia. Recent reviews have emphasized that dysfunction of the oral musculature represents an important but frequently under-recognized contributor to swallowing-related difficulties in individuals with facial paralysis [8].

A study conducted in a pediatric stroke population also reported that oral motor control, speech, and swallowing functions are closely associated through shared cranial nerve networks [17]. This finding may be consistent with the proposed neurophysiological relationship between oral motor impairment and swallowing function across different stroke populations. From a clinical perspective, these findings may suggest a potential association between oral motor impairments and swallowing-related difficulties in individuals with CFP. Therefore, detailed evaluation of oral motor components such as labial and buccal control may warrant consideration alongside traditional dysphagia assessments [18].

In addition, considering the role of saliva in bolus cohesion and oral cavity clearance [19], impairments in oral motor coordination may affect swallowing efficiency. Recent evidence has highlighted that facial motor dysfunction may adversely affect oral competence, saliva management, oral residue clearance, and bolus control, potentially contributing to swallowing-related difficulties [8]. These factors may warrant consideration in dysphagia management, particularly with regard to oral residue accumulation and inadequate oral clearance behaviors.

In our study, the close association between labial and buccal functions and oral motor dysfunction supports impairment in mastication organization and the oral preparatory phase. In contrast, the present study primarily focused on facial motor function, oral motor dysfunction, and swallowing-related measures. Because sensory involvement was not systematically assessed, the potential contribution of sensory factors to swallowing-related impairments remains unclear. The literature investigating oral functions affected by facial paralysis remains limited. Previous studies reporting that reduced muscle tone following stroke may impair mastication function [20,21] emphasize the importance of managing oral functions affected by hemisyndrome. Experimental studies conducted by Mazari et al. (2007) also demonstrated that loss of buccal support may reduce mastication efficiency [22]. Our findings are consistent with these experimental observations and may suggest a potential association between buccal function and oral preparatory phase performance. Clinically, these findings may indicate a potential association between oral preparatory impairments and swallowing-related difficulties. Rehabilitation approaches targeting oral preparation and mastication processes may therefore warrant further investigation in future studies.

The bilateral characteristics of swallowing abnormalities within the oral cavity may be explained by the bilateral cortical and bulbar representation of swallowing function [23]. One possible explanation for the widespread distribution of oral motor impairment observed in our cohort is the complex bilateral neural organization of swallowing-related motor control, as suggested by previous neurophysiological studies. This finding is consistent with previous neurophysiological studies supporting the bilateral neural organization of swallowing function.

One of the notable findings of the study was the independent association between velar function and FOIS levels. The velopharyngeal mechanism plays an important role in generating intraoral pressure, effective bolus transfer from the oral cavity to the pharynx, and maintenance of nasopharyngeal protection. In cases of velar insufficiency, reduced oral pressure generation and impaired bolus propulsion may negatively affect oral intake. The independent association between velar function and FOIS scores in our study suggests that oropharyngeal transfer efficiency may play an important role in functional oral intake. This finding is consistent with the findings of Clavé et al. (2006), who reported that impairments in oral and pharyngeal coordination may affect functional oral intake [24]. In addition, recent post-stroke dysphagia literature has reported that oral phase impairment may be characterized by inadequate labial closure, impaired buccal sulcus clearance, insufficient bolus formation, and reduced posterior bolus propulsion [6]. The persistence of the association between velar function and FOIS may indicate that oral feeding capacity is associated not only with oral preparatory skills but also with oropharyngeal transfer efficiency. Furthermore, FOIS should be considered a functional scale reflecting the individual’s current oral feeding performance rather than a purely physiological swallowing measure. Clinically, these findings may indicate a potential association between velopharyngeal function and functional oral intake. Therefore, velopharyngeal control may warrant consideration during assessment and rehabilitation planning; however, further studies are needed to confirm this relationship.

The observed associations between labial and velar functions and EAT-10 scores may suggest that subjective dysphagia perception may be particularly associated with loss of oral control and impaired velopharyngeal coordination. Previous studies have reported that inadequate lip closure may result in liquid leakage and impaired saliva control. Impaired oral bolus control may also affect the organization of the swallowing reflex and the timing of oropharyngeal transfer, leading individuals to perceive symptoms such as difficulty eating, sensation of food sticking, or the need for repeated swallowing more intensely [25]. Our findings may suggest that subjective dysphagia symptoms are associated with task-specific oral motor components. The loss of significance of Sunnybrook scores in the regression model may indicate that specific oral motor impairments could have a greater influence on symptom perception than overall facial motor function level. This finding is consistent with the view of Belafsky et al. (2008) that EAT-10 reflects functional swallowing difficulties perceived in daily life [11].

The independent associations of RSST performance with labial and tongue functions suggest that repetitive swallowing ability may be influenced by oral motor coordination and lingual transport mechanisms [26]. Previous studies have reported that lingual strength and motor control are associated with oral transit duration and that reduced tongue pressure may negatively affect bolus transfer and oral phase organization [27]. Our findings may indicate a potential association between specific oral motor components, particularly labial and lingual functions, and repetitive swallowing performance. In addition to lingual function, recent clinical studies have suggested that broader facial motor impairment may influence swallowing efficiency through its effects on oral competence, saliva management, and oral phase coordination [8].

From a clinical perspective, persistent weakness or motor coordination impairment involving the orbicularis oris and buccinator muscles may negatively affect both speech and swallowing performance. Particularly in older individuals and those with systemic diseases that may weaken the cough reflex, CFP should be considered an additional risk factor for dysphagia. Furthermore, these findings suggest that rehabilitation approaches targeting lingual motor control may be important for improving swallowing efficiency.

Recent large-scale studies have demonstrated that facial palsy is present in approximately 40–50% of stroke patients and may persist beyond the acute phase in a substantial proportion of individuals. Furthermore, facial palsy has been associated with impairments in eating, drinking, communication, and overall functional recovery, emphasizing the importance of early identification and rehabilitation of facial motor dysfunction following stroke [18,28].

This study has several limitations. First, because of its cross-sectional design, causal relationships cannot be established, and the findings should be interpreted as associations rather than evidence of causality. In addition, participants were recruited from a single tertiary referral center using a convenience sampling approach. The study population also demonstrated substantial heterogeneity with respect to stroke severity (NIHSS range: 0–41) and time since stroke (1–28 months). Although these variables were incorporated into the regression models as potential confounders, residual heterogeneity may still have influenced the observed associations and may limit the generalizability of the findings to broader stroke populations.

Second, the absence of instrumental swallowing assessments, such as videofluoroscopic swallowing study or fiberoptic endoscopic evaluation of swallowing, limited the objective characterization of swallowing physiology and prevented direct confirmation of aspiration or specific phase-related swallowing abnormalities. In addition, detailed neuroanatomical lesion characteristics were not systematically analyzed. Because lesion location may influence both facial motor impairment and swallowing function after stroke, residual confounding related to lesion topography cannot be excluded.

Third, the oral motor assessment form was developed by the researchers and did not undergo a comprehensive psychometric validation process. Although internal consistency and inter-rater reliability analyses demonstrated acceptable overall performance, the moderate single-measure ICC values indicate that some degree of observer-related variability may have been present. Therefore, findings related to individual oral motor ratings should be interpreted cautiously and regarded as exploratory clinical observations rather than definitive psychometric outcomes. Future validation studies involving larger and independent cohorts are needed.

Furthermore, because several outcome measures consisted of ordinal or semi-quantitative clinical scales, regression analyses were primarily exploratory and should not be interpreted as predictive models. In addition, NIHSS emerged as a dominant predictor in several regression models, suggesting that overall neurological severity may account for part of the observed associations between oral motor dysfunction and swallowing-related outcomes. Therefore, the independent contribution of specific oral motor variables should be interpreted with caution.

Several assessments were clinician-rated and performed during the same evaluation session. Consequently, shared measurement variance and rater-related bias cannot be completely excluded. The very strong correlations observed between some variables may partially reflect overlapping clinical constructs, shared clinician-based assessment characteristics, and the fact that several measures evaluated related aspects of oral motor and swallowing function. Moreover, conceptual overlap among certain oral motor variables may have contributed to the elevated VIF values and multicollinearity observed in some regression models. Therefore, individual regression coefficients, particularly for variables with elevated VIF values, should be interpreted cautiously and should not be considered stable independent effect estimates.

Nevertheless, despite these limitations, this study provides a multidimensional clinical evaluation of the relationships among facial motor function, oral motor dysfunction, and swallowing-related outcomes in individuals with post-stroke CFP and may serve as a basis for future hypothesis-driven investigations using validated assessment tools and instrumental swallowing measures.

## 5. Conclusions

This study identified significant associations between facial motor impairment, swallowing function, and oral motor dysfunction in individuals with post-stroke CFP. In particular, labial, buccal, lingual, and velar motor impairments were associated with subjective dysphagia symptoms, functional oral intake level, and clinical swallowing performance. These findings may suggest a potential association between oral motor dysfunction and swallowing-related impairments in individuals with post-stroke CFP. Oral motor assessment may warrant consideration as part of a comprehensive dysphagia evaluation; however, further studies are required to clarify the nature and direction of these relationships.

The observed associations highlight the potential clinical relevance of facial and oral motor assessment in the evaluation of swallowing-related dysfunction in this population. However, because of the cross-sectional and exploratory nature of the study, these findings should be interpreted cautiously and should not be considered evidence of causal or predictive relationships. Future studies using instrumental swallowing assessments and validated oral motor evaluation tools are needed to further clarify these relationships.

## Figures and Tables

**Table 1:** Demographic and Clinical Characteristics of Participants.

Variable	*n* (%)	Mean ± SD	Min–Max
**Gender**			
Female	41 (51.2)	–	–
Male	39 (48.8)	–	–
**Stroke Type**			
Ischemic	42 (52.5)	–	–
Hemorrhagic	38 (47.5)	–	–
**Stroke Side**			
Right hemisphere	38 (47.5)	–	–
Left hemisphere	41 (51.2)	–	–
Bilateral	1 (1.3)	–	–
**Clinical Variables**			
Age (years)	80	53.73 ± 15.73	18–79
NIH Stroke Scale (NIHSS)	80	14.61 ± 13.68	0–41
Time since stroke (months)	80	7.53 ± 6.61	1–28
Sunnybrook total score	80	59.79 ± 31.79	4–100
EAT-10 score	80	16.76 ± 11.37	0–38
Repetitive Saliva Swallowing Test (RSST)	80	5.83 ± 3.02	0–13
Functional Oral Intake Scale (FOIS)	80	4.21 ± 1.95	1–7
**Oral Motor Function Parameters**			
Labial function (0–8)	80	3.45 ± 2.70	0–8
Tongue function (0–8)	80	3.34 ± 3.12	0–8
Mandibular function (0–6)	80	1.89 ± 1.13	0–4
Velar function (0–4)	80	1.70 ± 1.14	0–4
Buccal control (0–4)	80	2.51 ± 1.40	0–4
OMD total score	80	12.89 ± 8.29	0–26

SD, standard deviation; NIHSS, National Institutes of Health Stroke Scale; EAT-10, Eating Assessment Tool-10; RSST, Repetitive Saliva Swallowing Test; FOIS, Functional Oral Intake Scale. Higher OMD scores indicate greater oral motor dysfunction.

**Table 2:** Correlation Analysis Between Facial Motor Function, Swallowing Measuresand Oral Motor Parameters.

Variables	1	2	3	4	5	6	7	8	9	10
**1. Sunnybrook**	1									
**2. EAT-10**	−0.954	1								
**3. RSST**	0.914	−0.899	1							
**4. FOIS**	0.915	−0.888	0.866	1						
**5. Labial**	−0.938	0.925	−0.885	−0.887	1					
**6. Tongue**	−0.888	0.867	−0.766	−0.848	0.849	1				
**7. Mandibular**	−0.651	0.608	−0.614	−0.654	0.622	0.675	1			
**8. Velar**	−0.612	0.657	−0.604	−0.661	0.567	0.506	0.417	1		
**9. Buccal Control**	−0.796	0.807	−0.766	−0.758	0.799	0.745	0.585	0.598	1	
**10. OMD Total**	−0.946	0.936	−0.871	−0.915	0.942	0.939	0.748	0.670	0.870	1

Values represent Spearman correlation coefficients (r). All correlations were statistically significant at *p* < 0.001. Abbreviations: RSST, Repetitive Saliva Swallowing Test; FOIS, Functional Oral Intake Scale; OMD, Oral Motor Dysfunction. Higher OMD scores indicate greater oral motor dysfunction.

**Table 3:** Significant Predictors Identified in Hierarchical Regression Analyses for RSST and EAT-10 Outcomes.

Outcome	Model	Variable	B	SE	β	t	*p*	VIF
**RSST**	Model 1	NIHSS	−0.152	0.034	−0.691	−4.520	<0.001	8.035
	Model 2	NIHSS	−0.084	0.038	−0.381	−2.208	0.031	10.167
		Labial Function	−0.384	0.156	−0.343	−2.463	0.016	8.544
		Tongue Function	0.238	0.112	0.246	2.114	0.038	5.958
**EAT-10**	Model 1	NIHSS	0.810	0.087	0.974	9.255	<0.001	8.035
	Model 2	NIHSS	0.458	0.088	0.551	5.199	<0.001	10.167
		Labial Function	1.120	0.360	0.266	3.109	0.003	8.544
		Velar Function	1.094	0.391	0.110	2.797	0.007	1.802
		Buccal Control	1.001	0.495	0.123	2.021	0.047	4.330

Abbreviations: RSST, Repetitive Saliva Swallowing Test; EAT-10, Eating Assessment Tool-10; NIHSS, National Institutes of Health Stroke Scale; SE, Standard Error; VIF, Variance Inflation Factor.

**Table 4:** Significant Predictors Identified in Hierarchical Ordinal Logistic Regression Analysis for FOIS.

Model	Variable	B	SE	Wald χ^2^	*p*	OR	95% CI
Model 1	Time since stroke	−0.296	0.119	6.131	0.013	0.744	0.589–0.940
Model 1	NIHSS	−0.224	0.061	13.330	<0.001	0.799	0.709–0.901
Model 2	Velar function	−1.030	0.294	12.256	<0.001	0.357	0.200–0.636

Abbreviations: FOIS, Functional Oral Intake Scale; NIHSS, National Institutes of Health Stroke Scale; SE, Standard Error; OR, Odds Ratio; CI, Confidence Interval. Model 1: sex, stroke type, stroke side, time since stroke, and NIHSS. Model 2: Model 1 variables plus oral motor dysfunction parameters.

## Data Availability

The data supporting the findings of this study are available from the corresponding author upon reasonable request.
